# Coverage-preserving sparsification of overlap graphs for long-read assembly

**DOI:** 10.1093/bioinformatics/btad124

**Published:** 2023-03-09

**Authors:** Chirag Jain

**Affiliations:** Department of Computational and Data Sciences, Indian Institute of Science, Bengaluru, Karnataka 560012, India

## Abstract

**Motivation:**

Read-overlap-based graph data structures play a central role in computing *de novo* genome assembly. Most long-read assemblers use Myers’s string graph model to sparsify overlap graphs. Graph sparsification improves assembly contiguity by removing spurious and redundant connections. However, a graph model must be coverage-preserving, i.e. it must ensure that there exist walks in the graph that spell all chromosomes, given sufficient sequencing coverage. This property becomes even more important for diploid genomes, polyploid genomes, and metagenomes where there is a risk of losing haplotype-specific information.

**Results:**

We develop a novel theoretical framework under which the coverage-preserving properties of a graph model can be analyzed. We first prove that de Bruijn graph and overlap graph models are guaranteed to be coverage-preserving. We next show that the standard string graph model lacks this guarantee. The latter result is consistent with prior work suggesting that removal of contained reads, i.e. the reads that are substrings of other reads, can lead to coverage gaps during string graph construction. Our experiments done using simulated long reads from HG002 human diploid genome show that 50 coverage gaps are introduced on average by ignoring contained reads from nanopore datasets. To remedy this, we propose practical heuristics that are well-supported by our theoretical results and are useful to decide which contained reads should be retained to avoid coverage gaps. Our method retains a small fraction of contained reads (1–2%) and closes majority of the coverage gaps.

**Availability and implementation:**

Source code is available through GitHub (https://github.com/at-cg/ContainX) and Zenodo with doi: 10.5281/zenodo.7687543.

## 1 Introduction

Accuracy of long reads has improved tremendously over the last 3 years (Wenger et al. 2019). For instance, PacBio HiFi sequencing technology yields consensus sequencing reads that are both long (averaging 10–25 kb) and highly accurate (averaging 99.8%). Similarly, modal raw read accuracy above 99% has been reported using reads from Oxford Nanopore Technology (ONT) sequencing (Sereika et al. 2022). Consequently, long-read technologies have shown the greatest promise in computing high-quality haplotype-resolved genome assemblies (Jarvis et al. 2022).

The objective of genome assembly is to reconstruct the original sequence from a large number of reads. Read-overlap-based assembly algorithms work by constructing an *overlap graph* where each vertex corresponds to a read, and edges represent suffix-prefix overlaps between the reads. Practical algorithms account for the following two key challenges while computing genome assembly. First, due to the presence of repetitive sequences, it is usually possible to reconstruct different genomes using the same input set of reads, each of which is fully consistent with the data (Medvedev and Pop 2021). Accordingly, problem formulations for genome assembly which seek a single genome reconstruction, e.g. by finding a Hamiltonian cycle in an overlap graph, or computing an Eulerian cycle in a de Bruijn graph, are not used in practice. Instead, assemblers compute contigs which are long, contiguous segments that, in principle, correspond to substrings of the source genome (Tomescu and Medvedev 2017). Second, the presence of repeats, variable-length reads and read errors also leads to too many edges in the overlap graph. This challenge is addressed by using graph sparsification heuristics that exclude vertices and edges which are likely to be redundant or false.

**Figure 1:**
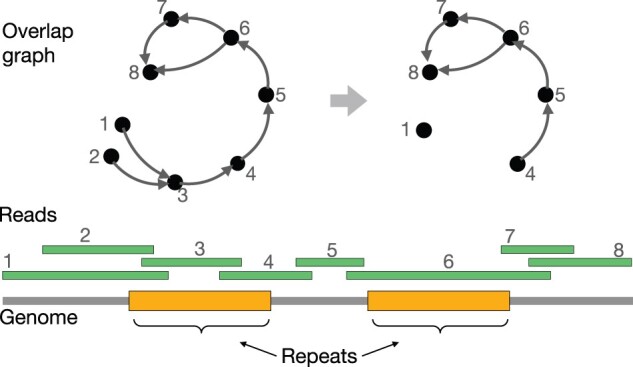
Illustration of an example where removal of vertices associated with contained reads leads to a coverage gap. Reads with IDs 2 and 3 are contained in 1 and 6, respectively. If the contained reads are ignored, there is no walk in the sparse graph that spells the genome

The commonly used *string graph* model by Myers (2005) (i) removes reads that are *contained* as a substring of a longer read (Fig. 1), (ii) ignores suffix-prefix overlaps that are shorter than the longest possible between a read pair, and (iii) removes transitive edges. More aggressive sparsification procedures also exist in the literature such as the *best overlap graph* approach of Miller et al. (2008) which retains only the best overlaps to a given read, where “best” is defined using some criterion. Despite the use of such graph sparsification techniques in most long-read assembly tools, theoretical understanding of these heuristics is fairly limited. A rigorous analysis of these sparsification techniques is important, especially in the context of diploid and polyploid genomes, because these can lead to loss of useful haplotype-specific information from an assembly graph. Shomorony et al. (2016) and Hui et al. (2016) gave provably efficient algorithms for overlap graph sparsification for fixed-length and variable-length reads, respectively. However, their formulations make a simplifying assumption that the input reads are long enough to avoid ambiguity caused by repeats, which may not hold in general.

Assuming a genome is sequenced with sufficient coverage, then there must exist walks in an assembly graph that can spell the true sequences corresponding to each chromosome. Due to repeats, these walks need not correspond to non-branching unitigs. An assembly graph which guarantees this property is said to be *coverage-preserving* (formally defined in Section 2). In this article, we make the following contributions:

1. We formulate the coverage-preserving property of genome assembly graph models, and perform a rigorous evaluation of three commonly used models: (i) de Bruijn graphs, (ii) overlap graphs, and (iii) string graphs. We show that de Bruijn graphs and overlap graphs are guaranteed to be coverage preserving, but string graphs are not.
2. Graph sparsification is a critical step during genome assembly to prune the overlap graph because it helps to compute longer contigs. We develop theoretical results to compute a sparse overlap graph while preserving the coverage-preserving property.
3. We extend the proposed theory into practical heuristics and a prototype software ContainX. The proposed heuristics are useful to identify redundant contained reads which can be excluded from overlap graph without violating the coverage-preserving property.
4. We conducted experiments by using long read datasets simulated from diploid HG002 human genome assembly while matching read length distributions to real PacBio HiFi and ONT data. We show that 1 and 50 coverage gaps are introduced on average by removing all contained reads in HiFi and ONT datasets, respectively. This is the first work to quantify the impact of graph sparsification heuristics in Myers’s string graph model.
5. For HG002 genome assembly, ContainX algorithm excludes 98–99% contained reads from the graphs and leaves ≤10% coverage gaps. This result compares favorably to the performance of other known solutions for this problem.

## 2 Concepts and notations

We set the stage by defining important notations and definitions. We will recall commonly used assembly graph models. Subsequently, we will give a formal definition of the desired coverage-preserving property in graphs.

### 2.1 Notation on strings

For a linear string $x=a_{1}\ldots a_{n}$ over alphabet Σ={A, C, G, T}, |x|=n is the length of *x*, $x[i]=a_{i}$ is the $i^{th}$ symbol of *X*, and $x[i:j]=a_{i}\ldots a_{j}$ is the substring from position *i* to position *j*. Let $x^{i}$ denote string *x* concatenated with itself *i* times. String *x* is said to have a suffix-prefix overlap of length *l* with string *y* when a proper suffix of length *l* in *x* equals a proper prefix of *y*. Recall that a proper prefix or a proper suffix of a string cannot be equal to the string itself.

We will use a circular genome model to avoid edge-effects associated with sequencing coverage. A circular string can be viewed as a traditional linear string which has the left- and the right-most symbols wrapped around and glued together. Circular string $z=\langle a_{1}\ldots a_{n}\rangle$ has length |z|=n. Substring z[i:j], where i∈[1,n],j≥i, equals the finite substring of the linear infinite string ${\left(a_{1}\ldots a_{n}\right)}^{\infty }$ from position *i* to position *j*. Note that offset *j* in z[i:j] can be greater than |*z*| because *z* is a circular string. Further, substring z[i:j] is said to be a *repetitive* string in *z* iff there exists $z[i^{\prime }:j^{\prime }]=z[i:j],i^{\prime }\neq i$. Suppose the true unknown genome is a set of circular strings, each representing a chromosome sequence. Let ϕ be a known upper bound on the maximum length of a chromosome in the genome.

A sequence *read* sampled from the genome is a substring of one of the circular strings in the genome. An indexed multiset of the reads is represented using symbol R. Read x∈R is labeled as a *contained* read if there exists a read y∈R∖{x} such that *x* is a substring of *y*. If *y* is also contained in *x*, then *x* and *y* are identical. In this case, break the tie by assuming that the read with lower index in R is contained within the read with higher index. The reads which contain read *x* are referred to as *parent* reads of *x*. We assume that either there are no sequencing errors or reads have been error-corrected. In our theoretical results, we will also assume that DNA is a single-stranded molecule.

### 2.2 Graph models for genome assembly

De Bruijn graphs, overlap graphs and string graphs are popular graph-based frameworks used for *de novo* genome assembly. Let G(V,E,σ,w) denote a directed assembly graph where vertices are labeled with strings, and edges indicate suffix-prefix overlap relations between the labels of connected vertices. Function $\sigma :V\to \Sigma^{+}$ assigns a string label to each vertex. Function w:E→N assigns a weight to each edge. Weight of an edge $v_{1}\to v_{2}$ signifies the length of the prefix of $\sigma (v_{1})$ that is not in the suffix-prefix match. In a de Bruijn graph $B_{k}(R)$, vertex set *V* corresponds to the set of all *k*-mer substrings in read set R, and an edge of weight one exists from vertex $v_{1}$ to vertex $v_{2}$ if and only if $\sigma (v_{1})[2:k]=\sigma (v_{2})[1:k-1]$ (Idury and Waterman 1995). This data structure is also sometimes referred to as node-centric de Bruijn graph (Chikhi and Rizk 2013).

Overlap graph O(R) is a directed multigraph where vertices correspond to reads, and edges correspond to suffix-prefix overlaps among the reads (Myers 1995). A directed edge $v_{1}\to v_{2}$ with weight $|\sigma (v_{1})|-l$ is drawn if and only if string $\sigma (v_{1})$ has a suffix-prefix overlap of length *l* with string $\sigma (v_{2})$. A subgraph of overlap graph O(R) that contains only the edges representing suffix-prefix overlaps of length ≥k is denoted as $O_{k}(R)$.

Myers (1995, 2005) introduced a sparse variant of overlap graph structure called as string graph. Unlike overlap graphs which are defined as directed multigraphs, string graph S(R) is a directed graph. A string graph includes only the longest suffix-prefix overlap between a read pair. Second, vertices associated with contained reads are excluded from a string graph. Finally, if vertex $v_{1}$ connects to $v_{2}$, $v_{2}$ connects to $v_{3}$ and $v_{1}$ connects to $v_{3}$ using edges $e_{1},e_{2}$ and $e_{3}$, respectively, and $w(e_{1})+w(e_{2})=w(e_{3})$, then edge $e_{3}$ is excluded from the string graph because it is transitively deducible. Removal of transitive edges is referred to as transitive sparsification. A subgraph of string graph S(R) which only uses suffix-prefix overlaps of length ≥k is denoted as $S_{k}(R)$. In practice, a string graph has significantly fewer edges compared to the overlap graph which enables computation of longer assembly contigs.

By performing a closed walk in an assembly graph, one can *spell* a circular string. This string is formed by concatenating labels of the vertices in the walk without spelling the overlapped substrings twice.

### 2.3 Coverage-preserving graph models

Unless all repeats can be unambiguously resolved, one can compute different genome reconstructions, each of which is fully consistent with input reads (Medvedev and Pop 2021). An assembly graph spells the true genome if it spells all the possible genome reconstructions.

**Figure 2:**
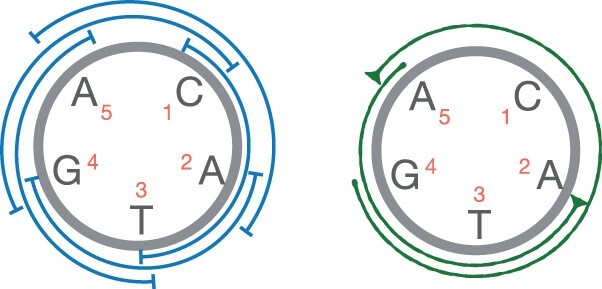
Suppose $l_{1}=4$ and $l_{2}=1$. A circular string z=〈CATGA〉 has five intervals of length $l_{1}-l_{2}=3$ as shown in blue. Two reads ACATG and ATGA, which match substrings z[5:9] and z[2:5], respectively, are shown in green. *z* has sufficient coverage because all five intervals include at least one of the starting positions of the reads

The depth of sequencing must be sufficient enough for assembling a genome (Lander and Waterman 1988). Informally, we will assume that reads “cover” the genome, and the length of suffix-prefix overlap between “consecutive” reads is above a certain threshold. One way to achieve this is following. We say that there is *sufficient coverage* over circular string *z* if there exist parameters $l_{1},l_{2}\in N$ such that $l_{1}>l_{2}$ and all intervals of length $l_{1}-l_{2}$ in *z* include the start of at least one substring of length $\geq l_{1}$ that matches a read ∈R. See Fig. 2 for an example. This definition supports variable-length reads and allows a read to match at multiple distinct places in a genome. We will frequently use this assumption later in our proofs in Section 3. Let $C(R,l_{1},l_{2},\phi )$ be the set of all *candidate* circular strings of length ≤ϕ which satisfy the stated coverage assumption using a given read set R and coverage parameters $\left(l_{1},l_{2}\right)$. The true genome is a subset of $C(R,l_{1},l_{2},\phi )$.

Definition Given a set of reads R, coverage parameters $\left(l_{1},l_{2}\right)$ and chromosome length threshold ϕ, graph G(V,E,σ,w) is said to be ***coverage-preserving*** if all candidate strings $\in C(R,l_{1},l_{2},\phi )$ can be spelled in the graph.

## 3 Analysis of known graph models

Theorem 1.  *de Bruijn graph* $B_{k}(R)$*is coverage-preserving for all* $k\leq l_{2}+1$.

Proof. If a circular string can be spelled in $B_{l_{2}+1}(R)$, then it can certainly be spelled in $B_{k}(R)$ for all $k<l_{2}+1$. We will prove that $B_{k=l_{2}+1}(R)$ is coverage-preserving by using contradiction. Suppose there exists a circular string $z\in C(R,l_{1},l_{2},\phi )$ which cannot be spelled in $B_{k}(R)$. Let $k_{i}\;(1\leq i\leq n)$ denote the *k*-mer substring starting from position *i* in *z*. If all $k_{i}$s exist in the set of all *k*-mer substrings extracted from read set R, then it is trivial to construct a closed walk in $B_{k}(R)$ which spells *z*. Therefore, at least one of the $k_{i}$s must be missing. Consider the minimum *i* for which this is true. Next, consider the $\left(l_{1}-l_{2}\right)$-long interval ending at the position *i* in the circular string *z*. Based on the coverage assumption, there is at least one read ∈R which matches a substring of length $\geq l_{1}$ of *z* starting in this interval. Such a read must contain *k*-mer $k_{i}$ as its substring. **□**

Next, we turn our attention to overlap graphs. Unlike de bruijn graphs, overlap graphs require a more careful analysis due to variable-length string labels on vertices and variable-length suffix-prefix overlaps. Our aim is to prove the following.

Theorem 2.  *Overlap graph* $O_{k}(R)$*is coverage-preserving for all* $k\leq l_{2}$.

If a circular string can be spelled in $O_{l_{2}}(R)$, then it can be spelled in $O_{k}(R)$ for all $k<l_{2}$. For a circular string $z\in C(R,l_{1},l_{2},\phi )$, we propose an algorithm to identify a closed walk in $O_{l_{2}}(R)$ which spells *z*. For this, we first need some definitions. Let $A_{i}$ be the $\left(l_{1}-l_{2}\right)$-long interval starting at position *i* in *z*, for all *i* ranging from 1 to |*z*|. For each interval $A_{i}$, let $z[A_{i}.s:A_{i}.e]$ be the substring of length $\geq l_{1}$ which starts in interval $A_{i}$ and matches a read ∈R. If there are multiple such substrings available, pick the ones with the least value of $A_{i}.s$, and then with the least value of $A_{i}.e$. Let $A_{i}.r\in R$ denote a read that matches substring $z[A_{i}.s:A_{i}.e]$. Suppose $A_{q},q\in [1,|z|]$ is the interval which ends at position $A_{1}.s-1$ if $A_{1}.s>1$ or else at position |*z*| if $A_{1}.s=1$. Recall that a circular string is equivalent to any of its cyclic rotated version, therefore, assume wlog that $\left|A_{1}.r|=\max_{i\in [1,|z|]}|A_{i}.r\right|$. If $|A_{1}.r|\geq |z|+l_{2}$, it is trivial to construct a valid closed walk by using single vertex corresponding to read $A_{1}.r$. In the following, we will assume that $|A_{i}.r|<|z|+l_{2}$ for all i∈[1,|z|]. The inequalities $l_{1}\leq |A_{i}.r|$ and $|A_{i}.r|<|z|+l_{2}$ imply that $l_{1}-l_{2}<|z|$. Also |z|>1 because $l_{1}-l_{2}>0$.

Lemma 1.  *For any two consecutive intervals* $\left(A_{i},A_{i+1}\right)$*in* $\left(A_{1},A_{2},\ldots ,A_{\left|z\right|}\right)$, $A_{i}.e\geq A_{i+1}.s+l_{2}-1$.

Proof. All intervals including $A_{i}$ and $A_{i+1}$ are of length $l_{1}-l_{2}$. $A_{i}.e-A_{i+1}.s$ equals its minimum value $l_{2}-1$ when (a) $A_{i}.s$ equals the first position in interval $A_{i}$, (b) $A_{i+1}.s$ equals the last position in interval $A_{i+1}$, and (c) the substring $z[A_{i}.s:A_{i}.e]$ has minimum possible length $l_{1}$.□

**Figure 3:**
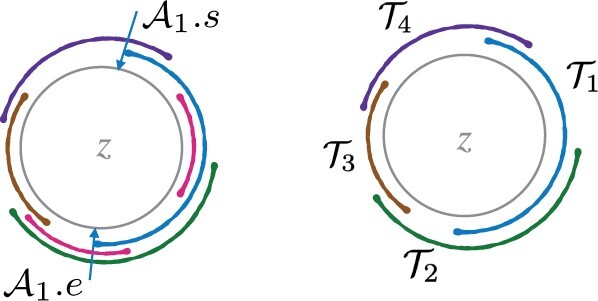
A graphic illustration of the subsequence computed from the complete sequence of intervals $A_{1},A_{2},\ldots ,A_{\left|z\right|}$. In the left figure, the gray-colored circle indicates circular string *z*, and the curved line segments indicate distinct substrings of *z* associated with intervals $A_{1},A_{2}\ldots ,A_{\left|z\right|}$. The right figure illustrates only the substrings associated with selected intervals in the subsequence $T_{1},T_{2},T_{3},T_{4}$

We will find a subsequence $\left(T_{1},T_{2},\ldots ,T_{p}\right)$ of $\left(A_{1},A_{2},\ldots ,A_{\left|z\right|}\right)$ such that vertices corresponding to reads $\left(T_{1}.r,T_{2}.r,\ldots ,T_{p}.r,T_{1}.r\right)$ can be connected to form a valid closed walk which spells *z* (Fig. 3). Let $T_{1}=A_{1}$. Suppose a partial subsequence $\left(T_{1},T_{2},\ldots ,T_{i}=A_{j}\right)$ has been computed so far. Continue by selecting the first interval among $\left(A_{j+1},\ldots ,A_{\left|z\right|}\right)$ as $T_{i+1}$ which satisfies the conditions $T_{i+1}.s>T_{i}.s$ and $T_{i+1}.e>T_{i}.e$. This selection procedure ensures that $T_{1}.s<T_{2}.s<\ldots <T_{p}.s$ and $T_{1}.e<T_{2}.e<\ldots <T_{p}.e$.

Lemma 2.  *Length of the subsequence* $\left(T_{1},T_{2},\ldots ,T_{p}\right)$*identified by the above procedure is at least two.*

Proof. By contradiction. Assume p=1, i.e. the computed subsequence is $T_{1}=A_{1}$. Based on our previous arguments, we have $|A_{1}.r|<|z|+l_{2}$, $l_{1}-l_{2}<|z|$ and |z|>1. Interval $A_{q}$ cannot contain position $A_{1}.s$ because size of each interval, i.e. $l_{1}-l_{2}$, is <|z|. Therefore, $A_{1}.s<A_{q}.s\leq |z|$. As interval $A_{q}$ was not picked among the subsequence of intervals, $A_{1}.e$ must be $\geq A_{q}.e$. Next, we will identify the minimum possible value of $A_{q}.e$ to get a lower bound of $A_{1}.e$. $A_{q}.e$ is lowest when the length of substring $z[A_{q}.s:A_{q}.e]$ is $l_{1}$ and $A_{q}.s$ equals the first position in $A_{q}$. Using these conditions, observe that $A_{q}.e\geq A_{1}.s+|z|+l_{2}-1$, and therefore, $A_{1}.e\geq A_{1}.s+|z|+l_{2}-1$. This implies that the length of substring $z[A_{1}.s:A_{1}.e]$ is $\geq |z|+l_{2}$. This is not possible when $|A_{1}.r|<|z|+l_{2}$. □

Lemma 3.  *For any two consecutive intervals* $\left(T_{i},T_{i+1}\right)$*in* $\left(T_{1},T_{2},\ldots ,T_{p}\right)$, $T_{i}.s<T_{i+1}.s$, $T_{i}.e<T_{i+1}.e$*and* $T_{i}.e\geq T_{i+1}.s+l_{2}-1$.

Proof. The first two inequalities $T_{i}.s<T_{i+1}.s$ and $T_{i}.e<T_{i+1}.e$ are guaranteed by the selection procedure of $T_{i+1}$. Suppose $T_{i}=A_{j}$ and $T_{i+1}=A_{k}$. From Lemma 1, we know that $A_{k-1}.e\geq A_{k}.s+l_{2}-1$. If j=k−1, then inequality $T_{i}.e\geq T_{i+1}.s+l_{2}-1$ holds. Otherwise, j<k−1 implies that $A_{k-1}$ was not picked among the subsequence of intervals. This would happen in either of the following two situations (i) $A_{j}.s=A_{k-1}.s,A_{j}.e=A_{k-1}.e$, (ii) $A_{j}.s<A_{k-1}.s,A_{j}.e\geq A_{k-1}.e$. In either case, $A_{j}.e\geq A_{k-1}.e$. Therefore, $T_{i}.e\geq T_{i+1}.s+l_{2}-1$. □

Lemma 4.
$T_{p}.s<T_{1}.s+|z|$ , $T_{p}.e<T_{1}.e+|z|$*and* $T_{p}.e\geq T_{1}.s+|z|+l_{2}-1$.

Proof. The first inequality $T_{p}.s<T_{1}.s+|z|$ holds because $T_{i}.s\in [1,|z|]\;\forall i\in [1,p]$. For the second inequality, recall that $\left|A_{1}.r|=\max_{i\in [1,|z|]}|A_{i}.r\right|$. If $T_{p}.e\geq T_{1}.e+|z|$, then $\left|A_{1}.r|<|T_{p}.r\right|$ which cannot be true. The third inequality can be proved by using the arguments from the proof of Lemma 2. If q<|z|, then the intervals $A_{q+1},\ldots ,A_{\left|z\right|}$ will contain $A_{1}.s$ and won’t be selected in the subsequence. As a result, either p=q or p<q. In either case, $T_{p}.e\geq A_{q}.e$. $A_{q}.e\geq A_{1}.s+|z|+l_{2}-1$ implies that $T_{p}.e\geq T_{1}.s+|z|+l_{2}-1$. □

Lemmas 3 and 4 collectively prove that vertices associated with the reads $T_{1}.r$, $T_{2}.r$, …, $T_{p}.r,T_{1}.r$ can be connected appropriately to build a valid closed walk in overlap graph $O_{l_{2}}(R)$. Along the walk, the nonoverlapping prefix of read $T_{i}.r\;(1\leq i<p)$ spells $z[T_{i}.s:T_{i+1}.s-1]$. The prefix of the last read $T_{p}.r$ spells $z[T_{p}.s:|z|]$. When $T_{1}.s>1$, it also spells $z[1:T_{1}.s-1]$. This completes the proof of Theorem 2. Finally, we consider the string graph model.

Observation. *String graph* $S_{k}(R)$*is not guaranteed to be coverage-preserving for any value of k*.

**Figure 4:**
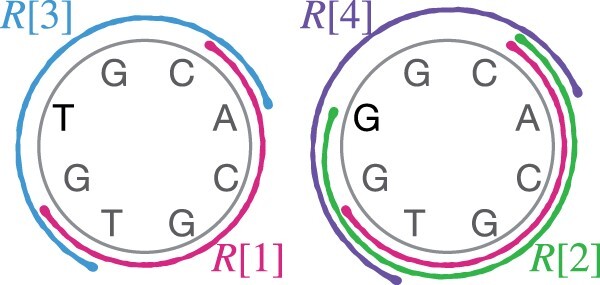
A visualization of the counter-example used to analyze the string graph model

A counter-example suffices to support the above statement (Fig. 4). Assume $l_{1}=6,l_{2}=2$ and ϕ=10. Suppose count of reads |R|=4,R[1]= CACGTG, R[2]= CACGTGG, R[3]= TGTGCA and R[4]= TGGGCA. Accordingly, the two candidate circular strings are 〈CACGTGTG〉 (covered by R[1],R[3]) and 〈CACGTGGG〉 (covered by R[1],R[2],R[4]). However, read R[1] is contained in read R[2] and the vertex corresponding to read R[1] is excluded from the string graph. As a result, the first candidate 〈CACGTGTG〉 cannot be spelled.

The significance of the above result is that despite sufficient sequencing coverage, string graphs may lose coverage over the genome. This limitation can lead to fragmented assembly in practice (Feng et al. 2022; Nurk et al. 2022). Accordingly, the following questions need to be addressed: (i) To what extent does the lack of the above guarantee affect quality of string-graph-based long-read assemblies in practice? and (ii) Does there exist an alternate graph model derived from an overlap graph which is as sparse as the string graph in practice, but guaranteed to be coverage-preserving?

## 4 An alternative framework for sparsification of overlap graphs

String graph is a subgraph of an overlap graph that loses the coverage-preserving guarantee after pruning selected vertices and edges. In this section, we propose techniques to compute a directed multigraph structure which is also a subgraph of overlap graph, and it is guaranteed to be coverage-preserving. Specifically, we propose *safe* graph sparsification rules for vertex and edge removal from overlap graph $O_{k}(R),k\leq l_{2}$ which ensure that all circular strings $\in C(R,l_{1},l_{2},\phi )$ can be spelled in the sparse graph. Suppose $O_{k}^{\prime }(R)$ equals a subgraph of $O_{k}(R)$ after performing a sequence of safe operations. Let $W_{\phi }(G)$ denote the set of circular strings of length ≤ϕ which can be spelled in graph *G*. From Theorem 2, we know that $C(R,l_{1},l_{2},\phi )\subseteq W_{\phi }(O_{k}(R))$. Therefore, the next three lemmas hold true.

Lemma 5.  *Vertex u can be safely removed from graph* $O_{k}^{\prime }(R)$*if* $W_{\phi }(O_{k}^{\prime }(R))=W_{\phi }(O_{k}^{\prime }(R)-u)$.

Lemma 6.  *Edge* $uv_{l}$*, i.e. the edge from vertex u to vertex v of weight l can be safely removed from graph* $O_{k}^{\prime }(R)$*if* $W_{\phi }(O_{k}^{\prime }(R))=W_{\phi }(O_{k}^{\prime }(R)-uv_{l})$.

Lemma 7  (Myers 2005). *If vertex* $v_{1}$*connects to* $v_{2}$, $v_{2}$*connects to* $v_{3}$*and* $v_{1}$*connects to* $v_{3}$*using edges* $e_{1},e_{2}$*and* $e_{3}$*respectively such that* $w(e_{1})+w(e_{2})=w(e_{3})$*in graph* $O_{k}^{\prime }(R)$*, then edge* $e_{3}$*can be safely removed.*

One needs to be careful while removing contained reads. The next lemma suggests a condition for when it is safe to remove a contained read.

Lemma 8.  *The vertex corresponding to a contained read* r∈R*can be safely removed from graph* $O_{k}^{\prime }(R)$*if all the following conditions are satisfied:*

1. *Read r is a substring of exactly one candidate circular string* $z\in C(R,l_{1},l_{2},\phi )$.
2. *Read r matches a non-repetitive substring of z.*
3. *Graph* $O_{k}^{\prime }(R)$*contains a vertex corresponding to a parent read of r.*

Proof. Let $r_{p}$ be a parent read of read *r* whose corresponding vertex exists in graph $O_{k}^{\prime }(R)$. Denote the vertices corresponding to reads *r* and $r_{p}$ as *u* and $u_{p}$ respectively. Recall that all reads are substrings of the genome, and the genome is a subset of $C(R,l_{1},l_{2},\phi )$. If *r* is a substring of exactly one circular string $z\in C(R,l_{1},l_{2},\phi )$, then $r_{p}$ must be a substring of *z* and no other circular string in $C(R,l_{1},l_{2},\phi )$. We need to show that *z* can still be spelled after removing vertex *u* from graph $O_{k}^{\prime }(R)$. Suppose $(v_{1},e_{1},v_{2},e_{2},\ldots ,v_{n-1},e_{n-1},v_{n}),\;n>1,v_{n}=v_{1}$ is a closed walk that includes vertex *u* to spell *z*. WLOG, assume that $v_{1}=u$. Note that neither of the vertices $v_{2},v_{3},\ldots ,v_{n-1}$ equal *u* because read *r* matches a non-repetitive substring of *z*. Since $v_{1}=u$, the first character of *z* is spelled by using the first character of *r*, and z[1  :  | r|] matches read *r*. Vertex $v_{1}=u$ is said to span interval $A_{1}$ of length |*r*| starting from position one in *z*. Similarly, vertex $v_{2}$ spans interval $A_{2}$ of length $\left|\sigma (v_{2})\right|$ starting from position $w(e_{1})+1$ in *z*. In general, each vertex $v_{i}$ in the closed walk spans a unique interval $A_{i}$ with starting position ∈[1,|z|]. Next, we will construct a new closed walk to spell *z* that uses vertex $u_{p}$ instead of vertex *u*. The interval spanned by vertex $u_{p}$ in *z*, say $A_{\mathrm{new}}$, can be uniquely identified because substring z[1:  |r|] is non-repetitive. $A_{\mathrm{new}}$ subsumes $A_{1}=A_{n}$ and possibly a few other adjacent intervals $A_{2},A_{3},\ldots$ and $A_{n-1},A_{n-2},\ldots$. All vertices associated with the subsumed intervals can be removed and replaced with $u_{p}$. With additional refinements, this procedure can be used to form a closed walk that spells *z* without using vertex *u*. □

One can easily generalize Lemma 8 to a case where the contained read and its parent read have consistent matches in more than one candidate circular string. We will use Lemma 8 in Section 5 to motivate a practical heuristic. Finally, based on all such safe rules, it would be ideal to construct a “minimal” sparse overlap graph. However, implementing these rules in practice is not trivial. Suppose all sequencing errors are corrected by using an appropriate error-correction algorithm, we need to make an informed estimation of $l_{1},l_{2}$ and ϕ parameters. Computing the set of closed walks $W_{\phi }(G)$ efficiently in large overlap graphs is also challenging.

## 5 A Proof-of-concept implementation

We build a practical algorithm for overlap graph sparsification that removes transitive edges by using the property in Lemma 7, and filters out a subset of contained reads by using heuristics that are inspired from Lemmas 5 and 8. Our implementation is currently designed for error-free long-read sequencing data sampled from both strands of DNA. Reverse complement of a string *x* is denoted as $\bar{x}$. Reverse complements are handled by adding two separate vertices for each read: first for the read as given and second for its reverse complement. If vertex *v* corresponds to a read in either its forward or reverse-complemented orientation, then $\bar{v}$ refers to the vertex corresponding to the read in the other orientation. Therefore, $\bar{\sigma (v)}=\sigma (\bar{v})$. Graph G(V,E,σ,w) satisfies the following two properties: (i) $\forall v\in V,\bar{v}\in V$, and (ii) for each edge e∈E, say from $v_{1}$ to $v_{2}$, an edge $\bar{v_{2}}\to \bar{v_{1}}$ of weight $\left|\sigma (v_{2})|-(|\sigma (v_{1})|-w(e)\right)$ belongs to *E*.

Our implementation requires the following inputs from a user for the initial graph construction: (i) error-free long reads, (ii) minimum overlap length threshold, (iii) exact suffix-prefix overlaps, and (iv) exact match coordinates of each contained read to each of its parent read. The minimum overlap length cutoff is set to 5 kb by default. For our experiments, we used minimap2 (Li 2018) to compute all-versus-all read alignments. Overlaps available from minimap2 were filtered to satisfy the alignment length and 100% identity constraints. The first step in our implementation is to build an overlap graph from the input suffix-prefix overlaps, and separately label reads as either contained or non-contained. For each contained read *r*, a set of 4-tuples is used to save match information with the parent reads. For instance, tuple (p,i,j,+) indicates that read *r* matches substring p[i:j] of read p∈R. Similarly, tuple (p,i,j,−) indicates that read *r* matches substring $\bar{p}[i:j]$, where $\bar{p}$ is the reverse complement of read p∈R.

### 5.1 Transitive sparsification of a directed multigraph

The transitive sparsification property in Lemma 7 is inspired from Myers’s string graph formulation. However, the linear O(|E|) expected time algorithm from Myers (2005) to label transitive edges is not applicable here because our graph is a multigraph, i.e. graph can have multiple edges between a pair of source and target vertices. Moreover, Myers’s runtime analysis uses the property that the number of irreducible edges per vertex is constant in expectation, which does not hold in the presence of contained reads. We use a simple $O(|E|D^{2})$ time procedure (Algorithm 1) where *D* is the maximum out-degree of a vertex in *G*. We did not explore faster algorithms here because Algorithm 1 uses a small fraction of the overall assembly runtime.


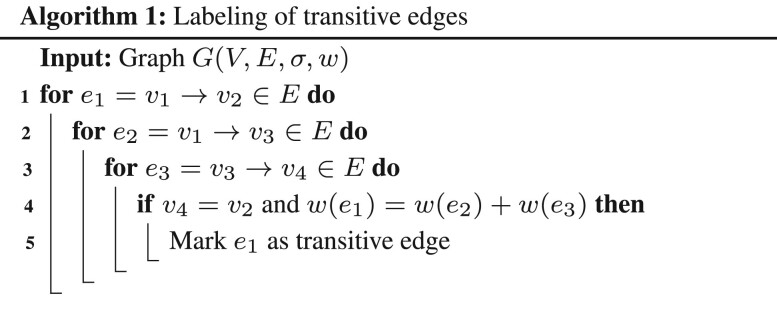


### 5.2 Remove non-repetitive haplotype-specific contained reads

**Figure 5:**
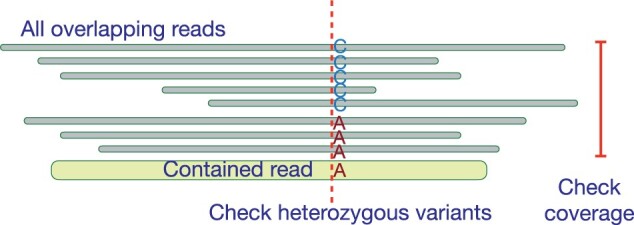
An illustration suggesting how multiple sequence alignment is used to detect non-repetitive haplotype-specific contained reads. We precompute the set of non-repetitive haplotype-specific reads by using a modified version of Hifiasm code (https://github.com/cjain7/hifiasm/tree/hifiasm_dev_debug)

Lemma 8 suggests that a contained read can be removed if (i) it is a substring of only a single haplotype, (ii) it is sampled from a non-repetitive genomic region, and (iii) at least one parent read is available in the graph. The third condition is automatically satisfied because there is at least one parent read of each read which is non-contained, and therefore, will not be removed. We propose a heuristic to check the first two conditions by computing all-versus-all read alignments using Hifiasm (Cheng et al. 2021). For each read *r*, we inspect multiple sequence alignment (MSA) of read *r* and other reads overlapping with read *r*. We check if the count of reads in the MSA does not greatly exceed the sequencing coverage to ensure that the read is sampled from a non-repetitive genomic region. For a diploid genome, we additionally check for the presence of a heterozygous variant using the MSA (Fig. 5). Presence of a heterozygous variant implies that read *r* aligns to a single haplotype. If both the conditions are satisfied, we remove the two vertices corresponding to contained read *r* and its reverse complement respectively.

### 5.3 *k*-mer-based filtering heuristic for contained reads

**Figure 6:**
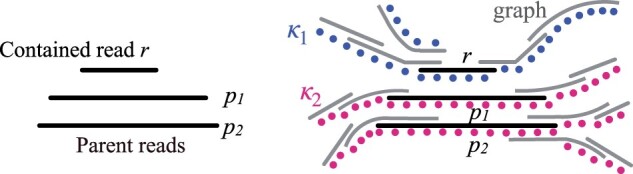
An illustration for how *k*-mer sets $\kappa_{1}$ and $\kappa_{2}$ are computed using bounded-length string walks from contained read *r* and its parent reads, respectively, in an overlap graph. Colored dots indicate the *k*-mers extracted from the strings

We process the remaining contained reads using a second filter. In theory, we can try enumerating all possible string walks with and without contained read. Comparing the two will inform whether the read can be safely removed (Lemma 5). However, enumerating all possible string walks at chromosome length scale is computationally prohibitive. We address this by implementing a *k*-mer-based heuristic. We first compute the set of *k*-mers observed in user-specified bounded-length string walks from the vertex associated with contained read (Fig. 6). By default, the length of these walks is set to twice the length of the contained read. The set of observed *k*-mers is denoted as $\kappa_{1}$. Next, we compute the union of set of *k*-mers observed in bounded-length string walks from the vertices associated with the parent reads of the contained read. This set is denoted as $\kappa_{2}$. If $\kappa_{1}\subseteq \kappa_{2}$, we mark the contained read for removal. This heuristic estimates if there exists a string which cannot be spelled after removal of the contained read.

## 6 Experimental results

Our evaluation addresses two questions: (a) How many coverage gaps are introduced if we follow the standard string graph model, i.e. discard all contained reads during graph construction?, and (b) How well does the proposed implementation (hereon referred to as ContainX) perform when compared to string graph as well as other existing methods?

### 6.1 Experimental setup

#### Read simulation from human haploid and diploid genomes

We used two human genome assemblies for read simulation. The first is a CHM13 haploid genome assembly provided by the Telomere-to-Telomere consortium (GenBank id GCA_009914755.4) (Nurk et al. 2022). This haploid genome assembly of size 3.1 Gbp has 25 contigs and N50 length 150.6 Mbp. The second assembly is a long-read diploid genome assembly (ftp://ftp.dfci.harvard.edu/pub/hli/hifiasm-phase/v2/HG002.hifiasm.trio.0.16.1.hap1.fa.gz and ftp://ftp.dfci.harvard.edu/pub/hli/hifiasm-phase/v2/HG002.hifiasm.trio.0.16.1.hap2.fa.gz) of HG002 human sample computed using Trio-hifiasm (Cheng et al. 2021). This diploid genome assembly of size 6.0 Gbp has 970 contigs and N50 length 57.8 Mbp. From these genome assemblies, we simulated eight error-free read sets whose length distributions are compatible with real PacBio HiFi and ONT sequencing data. We used Seqrequester (https://github.com/marbl/seqrequester) to simulate reads from random start positions in both forward and reverse orientations. Seqrequester allows users to specify a desired read length distribution. We used HiFi and ONT read length distributions from publicly-available long-read datasets of HG02080 human sample (Liao et al. 2022). Four long-read sets were simulated from haploid genome assembly with 20-fold coverage. The other four long-read sets were simulated from diploid genome assembly with 30-fold coverage (15-fold per haplotype). Length statistics and coverage information of the simulated read sets are shown in Table 1. The commands used to run the tools are listed in Supplementary Table S1.

**Table 1.** Properties of the simulated long-read sequencing datasets including their size and length statistics.

Dataset	Count of reads	N50 length	Max. length
HAPLOID-20x-ONT-1	3.7M	40 098	572 359
HAPLOID-20x-ONT-2	3.7M	40 089	544 992
HAPLOID-20x-HiFi-1	2.9M	21 314	48 708
HAPLOID-20x-HiFi-2	2.9M	21 310	48 708
DIPLOID-30x-ONT-1	5.3M	39 715	544 992
DIPLOID-30x-ONT-2	5.3M	39 732	572 359
DIPLOID-30x-HiFi-1	4.2M	21 295	48 708
DIPLOID-30x-HiFi-2	4.2M	21 298	48 708

### 6.2 Assessment of coverage gaps caused by removing contained reads

#### Benchmarking procedure

We used the following method to estimate the count of coverage gaps that are introduced by removal of contained reads. We computed all-versus-all read overlaps by using minimap2 v2.23 (Li 2018) to identify the set of contained reads. We labeled a read as contained if it matched a proper substring of a longer read. We can have false negatives because minimap2 uses *k*-mer-based heuristics. However, there cannot be false positives here because each overlap is verified using sequence alignment. In the second step, the set of non-contained reads was mapped to the genome. For the diploid genome, the set of non-contained reads was mapped to both paternal and maternal haplotypes, one by one. Minimap2 parameters were adjusted to enable reporting of multiple best alignments per read. Whenever a read aligned end-to-end against a genomic interval with 100% alignment identity, we recorded the interval. Finally, all the genomic intervals where no alignment coverage was observed were extracted using bedtools v2.29.1 (Quinlan and Hall 2010). Not all of these can be considered as coverage gaps caused by removal of contained reads. The sequencing coverage and read lengths at the two extreme ends of each contig are expected to be lower than the whole-genome average due to edge effect. Therefore, the intervals that overlap with either the first 25 kb or the last 25 kb bases of a contig are not considered. Similarly, the intervals that overlap with segments of the genome with zero sequencing coverage are also not considered. The remaining intervals are the coverage gaps introduced by removal of contained reads.

#### Results

The counts of coverage gaps observed in the four datasets are shown in Table 2. We observe that there are zero coverage gaps introduced in haploid genome for both ONT and HiFi read sets. In theory, haploid genomes can suffer coverage gaps due to identical repeats in the genome (Fig. 1). However, removal of contained reads does not cause any harm in practice. In diploid datasets, we observe 1 and 46–54 coverage gaps introduced in HiFi and ONT datasets, respectively. Compared to haploid genomes, coverage gaps in diploid genomes are more likely to happen because a longer read sampled from one haplotype can subsume all reads sampled from the homologous loci of the other haplotype, especially in regions with low heterozygosity. Moreover, ONT read length distribution is highly nonuniform which leads to a higher fraction of contained reads, and therefore, more coverage gaps. Supplementary Fig. S1 shows long-read length distributions.

**Table 2.** Coverage gap statistics for the gaps introduced by removal of contained reads.^a^

Dataset	Count of contained reads	Coverage gap statistics	
		Count	Max. length
HAPLOID-20x-ONT-1	3.2M	0	
HAPLOID-20x-ONT-2	3.2M	0	
HAPLOID-20x-HiFi-1	1.9M	0	
HAPLOID-20x-HiFi-2	1.9M	0	
DIPLOID-30x-ONT-1	4.6M	46	53 490
DIPLOID-30x-ONT-2	4.6M	54	101 371
DIPLOID-30x-HiFi-1	2.5M	1	1808
DIPLOID-30x-HiFi-2	2.5M	1	224

a Count of contained reads is estimated from all-to-all read alignments.

We further investigated whether the observed gaps are clustered in a particular chromosome, but we did not observe such behavior. The coordinates of these coverage gaps in the HG002 assembly were mapped to the coordinates of GRCh38 human genome reference by using paftools (Li 2018). The complete lists of these coordinates are provided in Supplementary Tables S2–S5. We also evaluated heterozygosity rate by checking the count of heterozygous variants in these gaps. These gaps collectively span 1.26 million bases on GRCh38 genome reference when all the four diploid datasets are considered together. However, Dipcall (Li et al. 2018) reported only 83 heterozygous variants. This rate is an order of magnitude lower relative to the whole-genome average heterozygosity rate 0.1% in HG002 genome. This observation confirms our expectation that the coverage gaps in a diploid genome are more likely to happen in the regions with low heterozygosity.

### 6.3 Evaluation of the proposed sparsification algorithm

#### Benchmarking procedure

We evaluated the performance of our proof-of-concept implementation ContainX that determines a subset of contained reads to be retained. We tested ContainX using the diploid datasets. We checked the performance by observing the following two parameters: (i) count of coverage gaps, and (ii) count of “junction” reads, i.e. the reads which correspond to vertices having either >1 incoming or >1 outgoing edges in the graph. Retaining more contained reads implies that their corresponding vertices and edges are retained in the graph. This may result in more number of junction reads which can further result in shorter unitigs. It is important to look at the two parameters simultaneously because the first parameter can be optimized by retaining all contained reads whereas the second parameter can be optimized by removing all contained reads. An ideal solution should optimize both by doing a careful selection of contained reads. To measure the first parameter, we used minimap2 to compute end-to-end 100% identical read alignments of the retained contained reads against the two haplotypes. We checked if these alignments closed the coverage gaps that were previously caused by discarding all contained reads.

#### Competing methods

We compared ContainX with four other methods. The first method, called as *Retain-all*, simply retains all contained reads. The second method, called as *Remove-all*, discards all contained reads. The third method is from Hui et al. (2016). It removes a contained read *r* if two of its parent reads are *inconsistent*. Two parent reads are said to be inconsistent if, when aligned with respect to read *r*, they disagree at some base. We also included Hifiasm in our benchmark. Unlike previous methods which stop at graph construction, Hifiasm is a full-fledged genome assembler that incorporates multiple heuristics (e.g. tip removal, bubble popping etc.) for graph sparsification. Hifiasm builds its initial overlap graph without using contained reads. It uses a heuristic to recall a few selected contained reads which can connect ends of two unitigs. Even though Hifiasm is specifically designed for HiFi reads, we tested it on ONT datasets as well because of error-free read simulation.

#### Results

In all four datasets, Retain-all method retained the highest number of contained reads as expected (Table 3). It resolved all coverage gaps, but the corresponding graph has significantly higher number of junction reads compared to Remove-all. Next, Hui-2016 filtering method appears to be conservative, i.e. it typically ends up retaining a majority of contained reads. In several cases, the contained read may have parent reads which agree at all bases (e.g. when they come from a homozygous region of the genome). There may also be cases when there is only a single parent read available. In all such scenarios, Hui-2016 heuristic will choose to retain the contained reads. This heuristic resolved all the coverage gaps. Remove-all method has the fewest junction reads among the four methods but has the highest number of unresolved coverage gaps as expected.

**Table 3.** Performance comparison of ContainX with four other methods.^a^

Dataset	Method	Retained contained reads	Junction reads	Coverage gaps
DIPLOID-30x-ONT-1	Retain-all	2.8M	2.5M	0
	Hui-2016	2.5M	2.3M	0
	ContainX	28.5K	53.9K	2
	Hifiasm	4.0K	1.7K	33
	Remove-all	0	38.9K	46
DIPLOID-30x-ONT-2	Retain-all	2.8M	2.5M	0
	Hui-2016	2.5M	2.3M	0
	ContainX	28.4K	53.8K	5
	Hifiasm	3.7K	1.7K	39
	Remove-all	0	38.5K	54
DIPLOID-30x-HiFi-1	Retain-all	2.5M	3.4M	0
	Hui-2016	2.5M	3.3M	0
	ContainX	39.8K	184.1K	0
	Hifiasm	164	36.9K	0
	Remove-all	0	158.4K	1
DIPLOID-30x-HiFi-2	Retain-all	2.5M	3.4M	0
	Hui-2016	2.5M	3.3M	0
	ContainX	39.9K	184.6K	0
	Hifiasm	149	37.2K	1
	Remove-all	0	158.5K	1

a Symbol “K” means thousand and “M” means million.

ContainX delivered favorable performance compared to the three methods. Using both datasets, ContainX retained 1–2% contained reads compared to Retain-all, yet it successfully resolved majority of the coverage gaps. ContainX resulted in only 2–5% junction reads when compared to Retain-all. This suggests that ContainX heuristics are promising in terms of improving assembly quality by avoiding coverage gaps. The count of false positives, i.e. the count of redundant contained reads retained by ContainX, is still notable. Most of these false positives correspond to contained reads that are sampled from long near-identical repetitive regions (Supplementary Fig. S2). This is a limitation of our *k*-mer-based filtering heuristic (Section 5.3) because it may retain redundant contained reads if it finds neighboring vertices that correspond to paralogous sequences during graph traversal. It is unclear whether it is possible to remove such contained reads with a provably-good graph-theoretic strategy. In some other cases, a redundant contained read may be retained if it has suffix-prefix overlaps with reads from both haplotypes while its parent reads correspond to a single haplotype. Finally, Hifiasm retained fewer contained reads than ContainX but it failed to resolve a majority of coverage gaps. This suggests that there is further scope to improve Hifiasm algorithm. The unitig graph of Hifiasm has the least number of junction reads because it does additional graph pruning which is necessary for computing longer unitigs. Incorporating ContainX heuristics inside Hifiasm code can be an interesting direction to explore.

The proposed graph sparsification algorithms implemented in ContainX are easy-to-implement, fast and space-efficient. The *k*-mer-based heuristic (Section 5.3) is parallelized by considering each contained read independently. The time required for this step on a multicore AMD processor is about ten minutes. Our other heuristic that detects heterozygous variants by using MSA of overlapping reads (Section 5.2) can be assumed to incur no additional time because this step is a default requirement in overlap-graph-based genome assemblers. In summary, the time needed by the proposed heuristics is insignificant compared to the main time-consuming step of computing all-versus-all read alignments in long-read assemblers.

## 7 Discussion

This work formalized the coverage-preserving property in assembly graph models. Development of provably-good graph models will help automate the computation of high-quality haplotype-resolved assemblies of personal human genomes (Jarvis et al. 2022). We showed a rigorous analysis of de Bruijn graph, overlap graph and string graph assembly models. We concluded that de Bruijn graph and overlap graph models offer the guarantee, but string graph model lacks this guarantee. This limitation of the string graph model can lead to fragmented assembly in practice (Feng et al. 2022; Nurk et al. 2022). Several graph sparsification heuristics, such as removal of contained reads, may be deemed safe at first due to a false intuition, e.g. it may appear that contained reads lack useful information for assembly. We have shown that contained reads should be assessed carefully to avoid suboptimal genome assembly. It may be possible that an optimally constructed sparse overlap graph is more tangled than a string graph, however, further graph simplification can be achieved with known techniques, e.g. aligning complementary sequencing data to the graph (Garg et al. 2018; Cheng et al. 2022; Rautiainen et al. 2023).

We quantified the count of coverage gaps in a real haploid and diploid genome assembly, respectively, through our experiments done using simulated long reads. Based on these observations, we expect coverage gaps to occur even more frequently in polyploid genomes and metagenomes. These results are expected to inspire further research to develop alternative models and sparsification procedures for assembly graphs. We conducted our experiments by using error-free long reads to quantify the coverage gaps. If real data is considered, one would need to preprocess reads to correct errors by computing consensus among the overlapping reads (Cheng et al. 2021; Bankevich et al. 2022), but error-correction methods are not necessarily perfect. Our analysis with error-free reads is useful to explicitly evaluate the graph sparsification algorithms. When errors are considered, additional coverage gaps may occur due to incorrect bases in the error-corrected reads (Fu et al. 2019; Zhang et al. 2020). Depending on the error-rate in the corrected reads, one would also need to adjust the definition of a contained read as a substring of another read while allowing a bounded number of edits (Myers 2005). This relaxation can lead to a more aggressive sparsification in string graphs, and therefore, more coverage gaps.

We implemented novel heuristics for assessing contained reads in ContainX that were motivated from our safe graph sparsification policies. We compared ContainX to heuristics from Hifiasm, Hui et al. (2016) and two other extreme policies that remove and retain all the contained reads respectively. None of these methods is able to simultaneously eliminate all gaps and all the redundant contained reads. ContainX provides favorable performance by eliminating most of the redundant contained reads and resolving majority of the coverage gaps. Our future work will explore integration of ContainX heuristics in long-read assemblers. Extending the proposed heuristics to erroneous reads should not be challenging. For example, one can still detect haplotype-specific contained reads by considering MSA of overlapping reads and ignoring variants that are not supported by sufficient number of reads (Cheng et al. 2021). Similarly, the proposed *k*-mer-based filtering heuristic would be applicable if low-frequency *k*-mers are ignored.

## Supplementary Material

btad124_Supplementary_Data
