# Supplementary material for: Coverage-preserving sparsification of overlap graphs for long-read assembly
Source: Bioinformatics. 2023 Mar 9;39(3):btad124. doi: 10.1093/bioinformatics/btad124 (PMC10132763; doi:10.1093/bioinformatics/btad124)
Supplement: btad124_Supplementary_Data [file btad124_supplementary_data.pdf]

Supplementary Information

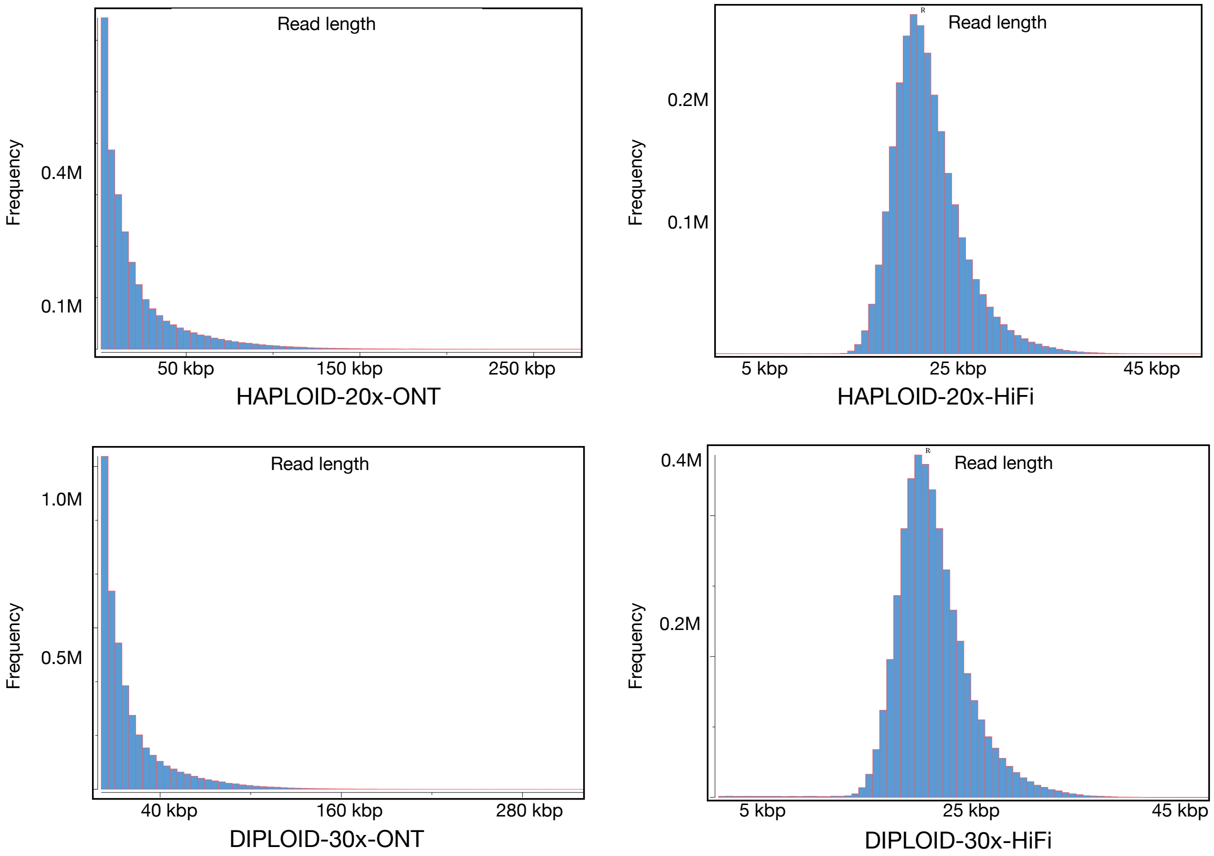

Fig. 1: Histogram of the number of reads with each read length. Reads of length < 1000 bp are not considered. The above plots show that read length distribution of ONT datasets is highly skewed with a large number of reads of short length. HiFi read lengths are within a narrow range compared to ONT. These are known characteristics of the two sequencing technologies.

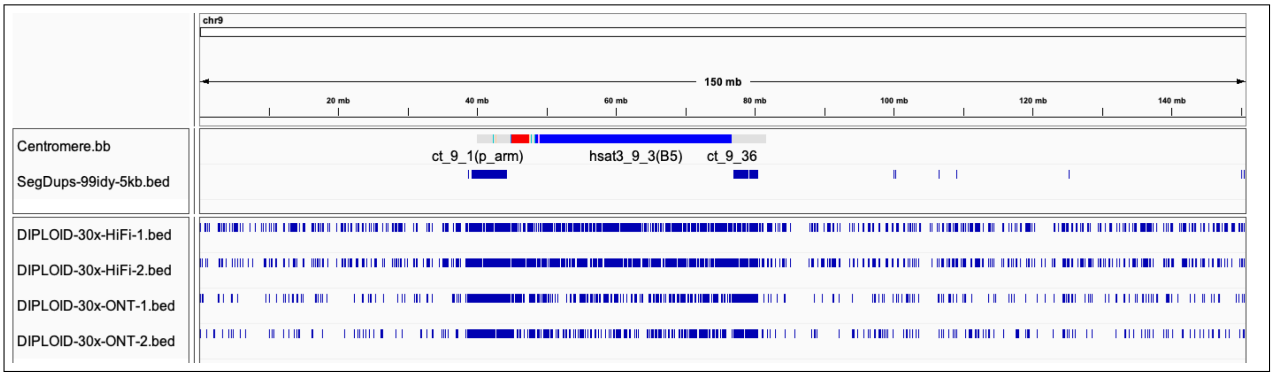

(a) Retained contained reads aligned to CHM13 chromosome 9

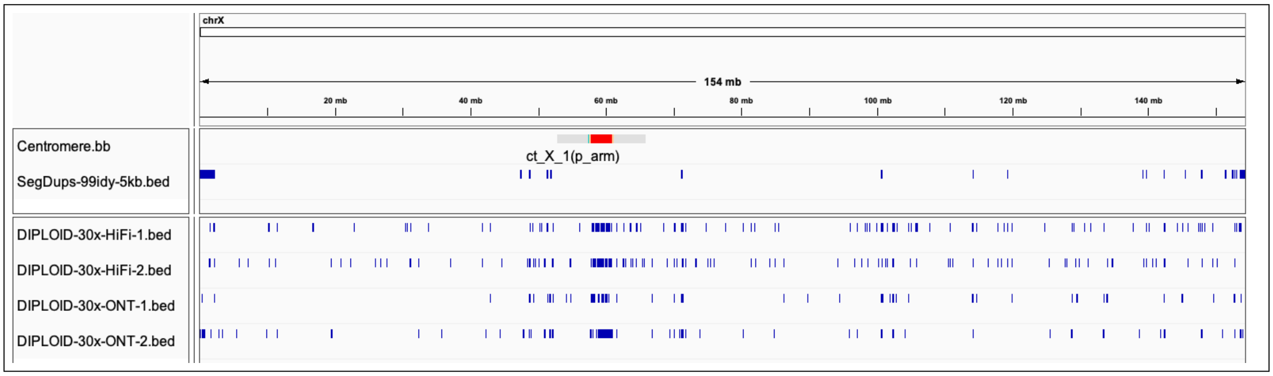

(b) Retained contained reads aligned to CHM13 chromosome X

Fig. 2: IGV visualisation of alignments of the contained reads retained by ContainX to CHM13v2.0 Telomere-to-Telomere assembly (GCA_009914755.4). The alignments were computed using minimap2 v2.23. We show a subset of alignments within chromosomes 9 and X. The above visualization is useful to understand why ContainX heuristics retain a large number of redundant contained reads in all four HG002 diploid datasets (see Table 3 in main text). The top two tracks in both figures show annotations for centromeres and segmental duplications (identity $\geq$ 99%, length $\geq$ 5kbp). These annotations were downloaded from UCSC Genome Browser. A significant number of read alignments overlap with centromeric and segmentally duplicated regions. We conclude that majority of ContainX false positives occur due to contained reads sampled from near-identical repeats of the genome.

<table>
<tr><th>Tool</th><th>Purpose</th><th>Commands</th></tr>
<tr><td>seqrequester (commit:1ee6fd9)</td><td>Read simulation</td><td><code>seqrequester simulate -truncate -genome HG002.hap1.fa -genomesize 2935689000 -coverage 15 -distribution histogram.txt</code></td></tr>
<tr><td rowspan="2">minimap2 (v2.23)</td><td>All-vs-all read alignments</td><td><code>minimap2 -t 32 -w 101 -k 27 -g 500 -B 8 -O 8,48 -E 4,2 -cx ava-ont reads.fasta reads.fasta > overlaps.paf</code></td></tr>
<tr><td>Read mapping</td><td><code>-t 32 -N 50 -cx map-ont reference.fasta reads.fasta > mm2.paf</code></td></tr>
<tr><td rowspan="4">ContainX

(commit:23e1feb)</td><td>Run ContainX</td><td><code>ContainX -p hetreads.txt -d dump.gfa -t 32 -n usefulContainedReads.txt reads.fasta overlaps.paf</code></td></tr>
<tr><td>Run Retain-all</td><td><code>ContainX -I 0 -d dump.gfa -t 32 -n usefulContainedReads.txt reads.fasta overlaps.paf</code></td></tr>
<tr><td>Run Remove-all</td><td><code>ContainX -I 0 -c -d dump.gfa -t 32 -n usefulContainedReads.txt reads.fasta overlaps.paf</code></td></tr>
<tr><td>Run Hui-2016</td><td><code>ContainX -h -d dump.gfa -t 32 -n usefulContainedReads.txt reads.fasta overlaps.paf</code></td></tr>
<tr><td>hifiasm (v0.16.1)</td><td>Test hifiasm</td><td><code>hifiasm -o output --write-paf -t 32 reads.fasta</code></td></tr>
<tr><td>Modified hifiasm (commit:59bcfd5)</td><td>For ContainX heuristic</td><td><code>hifiasm --dbg-het-cnt -o output --write-paf -t 32 reads.fasta</code></td></tr>
<tr><th colspan="2">Purpose</th><th>Commands</th></tr>
<tr><td colspan="2">Fetch contained read IDs from all-vs-all read alignments</td><td><code>cat overlaps.paf | awk '{if ($3 == 0 && $2 == $4 && $2 < $7 && $10 == $11) print $0}'> tmp
cat overlaps.paf | awk '{if ($8 == 0 && $7 == $9 && $7 < $2 && $10 == $11) print $0}'>> tmp
cat tmp | sort | uniq > containedIDs.txt</code></td></tr>
<tr><td colspan="2">Fetch contained read IDs used in hifiasm unitig graph</td><td><code>grep -P "^A\t" output.bp.r_utg.gfa | grep -o -P "read=[^\t]*" | sort > hifiasm.readIDs.txt
comm -12 hifiasm.readIDs.txt containedIDs.txt > hifiasm.containedreadIDs.txt</code></td></tr>
<tr><td colspan="2">Fetch count of junction reads in hifiasm unitig graph</td><td><code>grep "^L" output.bp.r_utg.gfa | cut -f2 | sort | uniq -c | grep -v "1 " | wc -l</code></td></tr>
<tr><td colspan="2">Fetch haplotype-specific non-repetitive read IDs using modified hifiasm code</td><td><code>cat output.het_cnt.log | tr -d ">" | awk '{if ($2 > 0) {print $1}}'> selectedReadIDs.txt</code></td></tr>
</table>

Table 1: Some important commands and tools that were used in this study. Bash script files are also provided along with containX code.

HG002 genome assembly			GRCh38 genome reference		
Contig id	Start	End	Chromosome id	Start	End
h1tg000009l	110900070	110905875	chr1	117037725	117043529
h2tg000022l	15152870	15160560	chr1	213447093	213454782
h1tg000068l	2866076	2870654	chr1	231372139	231376767
h2tg000043l	14918403	14921887	chr2	73264698	73268182
h2tg000043l	2156839	2174983	chr2	86011544	86029687
h2tg000023l	7722607	7738066	chr3	7727629	7743090
h2tg000010l	82369634	82395207	chr3	114613975	114639545
h2tg000034l	15546170	15547725	chr4	24679008	24680563
h2tg000014l	98646906	98657209	chr4	84170623	84180925
h2tg000014l	21807903	21814254	chr4	161020948	161027327
h2tg000017l	10313522	10314556	chr5	164430844	164431883
h1tg000006l	20492323	20506277	chr6	20516506	20530473
h1tg000036l	62513086	62523058	chr6	95294581	95304553
h1tg000036l	24475930	24479551	chr6	133346301	133349923
h1tg000036l	21309213	21329934	chr6	136496415	136517130
h1tg000036l	8446464	8499954	chr6	149308719	149362388
h2tg000049l	9904947	9912852	chr7	24201066	24208971
h1tg000010l	26527715	26546509	chr7	27410220	27429019
h1tg000067l	14144866	14160305	chr7	86898659	86914094
h1tg000023l	40382649	40397054	chr7	141372589	141386990
h1tg000003l	74679982	74689676	chr8	76860952	76870646
h2tg000011l	53131095	53153351	chr8	97022436	97044663
h1tg000021l	31311351	31311574	chr8	109880707	109880930
h2tg000001l	20933873	20947186	chr10	63801174	63811239
h2tg000001l	20933873	20947186	chr10	63811868	63813558
h1tg000007l	39009214	39010750	chr10	77733511	77735047
h2tg000001l	53657444	53660889	chr10	96441169	96444614
h1tg000032l	26710243	26711572	chr11	108418486	108419815
h1tg000032l	24316455	24321327	chr11	110809522	110814394
h1tg000026l	81896783	81946294	chr12	41557194	41606710
h1tg000026l	69278071	69293106	chr12	54217901	54232937
h2tg000004l	17612446	17614118	chr12	54572215	54573889
h1tg000026l	39760070	39761374	chr12	83726976	83728279
h1tg000026l	38832150	38833350	chr12	84657866	84659066
h1tg000017l	10534178	10543269	chr14	30228290	30237381
h2tg000003l	81495165	81500085	chr14	100294778	100299697
h1tg000017l	83021339	83027909	chr14	102667673	102674243
h2tg000002l	49073036	49081755	chr15	28042576	28051295
h2tg000002l	48832864	48844715	chr15	28279362	28291210
h1tg000005l	46151572	46153525	chr15	30938120	30940068
h2tg000002l	15585368	15615044	chr15	61449613	61479284
h1tg000123l	5886845	5887779	chr16	9342670	9343604
h2tg000057l	199241	206544	chr17	6652296	6659599
h1tg000027l	14645437	14651469	chr20	14625327	14631355
h2tg000059l	15875485	15884831	chr21	28368387	28377734
h1tg000064l	15871721	15877214	chr22	34851127	34856633

Table 2: Start–stop coordinates of coverage gaps caused by removing contained reads in DIPLOID-30x-ONT-1 dataset. The coordinates in HG002 assembly are also translated (i.e., lifted over) to GRCh38 genome reference.

HG002 genome assembly			GRCh38 genome reference		
Contig id	Start	End	Chromosome id	Start	End
h1tg000009l	18493205	18504097	chr1	24689133	24700023
h1tg000009l	86945009	86955138	chr1	93087776	93097904
h1tg000009l	103508009	103514037	chr1	109640847	109646875
h1tg000012l	64787435	64888806	chr1	214650325	214751706
h1tg000081l	4756106	4770890	chr2	73204290	73219075
h1tg000081l	11019519	11024843	chr2	79484322	79489661
h2tg000025l	52097255	52097889	chr2	212663821	212664455
h1tg000001l	92817961	92818455	chr2	223349132	223349626
h1tg000004l	8873984	8877229	chr3	8874669	8877914
h2tg000184l	232777	248699	chr3	74871732	74887654
h2tg000034l	4797508	4823424	chr4	13932709	13958691
h1tg000042l	114230605	114234253	chr4	73904437	73908085
h1tg000042l	104113807	104122161	chr4	84022694	84031047
h2tg000014l	92028013	92033684	chr4	90811125	90816796
h2tg000014l	82459823	82460036	chr4	100390877	100391090
h2tg000014l	70702729	70707859	chr4	112148705	112153851
h1tg000042l	15091152	15118100	chr4	173050753	173077668
h1tg000016l	91734766	91742004	chr5	83043812	83051050
h2tg000017l	90073347	90073761	chr5	84683858	84684272
h1tg000006l	20609242	20654818	chr6	20633454	20679068
h2tg000018l	25740048	25747436	chr6	33724209	33731597
h1tg000006l	50115047	50118074	chr6	50067746	50070778
h2tg000021l	5153975	5157462	chr6	64965355	64968838
h1tg000036l	90457178	90459095	chr6	67337810	67339727
h1tg000010l	20186319	20234628	chr7	21071775	21120082
h2tg000049l	12785813	12787547	chr7	21326099	21327801
h2tg000049l	9850119	9868340	chr7	24245575	24263817
h1tg000145l	1110399	1110479	chr7	67358338	67358418
h2tg000006l	26473188	26544931	chr7	132862940	132934640
h2tg000006l	26231773	26253441	chr7	133155335	133177002
h1tg000023l	40495078	40505896	chr7	141485319	141496136
h1tg000003l	46208131	46209187	chr8	48451474	48452530
h1tg000003l	64446587	64457392	chr8	66634485	66645289
h2tg000011l	22611658	22627754	chr8	67277359	67293455
h1tg000059l	13139707	13143077	chr9	28243394	28246761
h2tg000041l	37246637	37249901	chr9	37218351	37221615
h1tg000007l	11801161	11811351	chr10	104918759	104928949
h2tg000038l	50521189	50532240	chr11	39142190	39153262
h1tg000011l	44597468	44597862	chr11	46713582	46713976
h2tg000007l	24589917	24627125	chr11	110494690	110531902
h2tg000037l	1789315	1816591	chr13	19277044	19304307
h2tg000037l	12969569	12969897	chr13	30439954	30440282
h2tg000037l	19506191	19509196	chr13	36983851	36986858
h1tg000022l	42499978	42505754	chr13	44248785	44254562
h2tg000037l	30583828	30599471	chr13	48052070	48067716
h2tg000037l	33124120	33126064	chr13	50591608	50593552
h1tg000033l	20360199	20363658	chr13	107116486	107119945
h2tg000003l	26788478	26790995	chr14	45599428	45601940
h2tg000002l	2549333	2575965	chr15	74425030	74451662
h1tg000005l	1570767	1570875	chr15	75451659	75451767
h1tg000069l	22949634	22972649	chr16	78611705	78634697
h1tg000030l	14192878	14193075	chr17	3288793	3288990
h2tg000224l	30443	72254	chr19	43484700	43525330
h1tg000060l	29858829	29903277	chr21	14516790	14561239

Table 3: Start–stop coordinates of coverage gaps caused by removing contained reads in DIPLOID-30x-ONT-2 dataset.

HG002 genome assembly			GRCh38 genome reference		
Contig id	Start	End	Chromosome id	Start	End
h1tg000036l	57223158	57224966	chr6	100588432	100590240

Table 4: Start–stop coordinates of coverage gaps caused by removing contained reads in DIPLOID-30x-HiFi-1 dataset.

HG002 genome assembly			GRCh38 genome reference		
Contig id	Start	End	Chromosome id	Start	End
h2tg000002l	32863923	32864147	chr15	44197800	44198024

Table 5: Start–stop coordinates of coverage gaps caused by removing contained reads in DIPLOID-30x-HiFi-2 dataset.
